# Exploring the diversity of three Northern Atlantic sea beet populations

**DOI:** 10.3389/fpls.2025.1635602

**Published:** 2025-08-14

**Authors:** Lisa Bertram, Mahmood Gholami, Friedrich Kopisch-Obuch, Matthias Frisch

**Affiliations:** ^1^ Institute of Agronomy and Plant Breeding II, Justus Liebig University, Giessen, Germany; ^2^ KWS SAAT SE & Co. KGaA, Einbeck, Germany

**Keywords:** *Beta vulgaris* ssp. *maritima*, beta maritima, sea beet, crop wild relatives (CWR), genetic diversity, population structure, association mapping

## Abstract

Sea beet [*Beta vulgaris* ssp. *maritima* (L.) Arcang.] populations exhibit high genetic diversity and possess valuable traits that could enhance resilience, disease resistance, and tolerance to abiotic stress. In contrast, the genetic variation in the sugar beet breeding gene pool is limited. Our objectives were to examine sea beet population diversity using high-density marker data and substantial sample sizes within a population simultaneously. Their genetic diversity and population structure were investigated to evaluate their potential for direct mapping of traits with association mapping. Within this study, a total of 1,363 genotypes across three Northern Atlantic sea beet populations from Denmark, France, and Ireland were analyzed using 16,201 SNP markers. The findings reveal genetic variation among the populations, with the Irish population exhibiting the highest genetic diversity and pronounced population structure. The Danish population showed low genetic diversity and minimal population structure, while the French population displayed intermediate levels of both, genetic diversity and population structure. Based on its high genetic diversity, the Irish population appears to have the most potential for use to directly map traits by association mapping, provided that the challenges posed by the severe population structure can be adequately addressed within analysis.

## 1 Introduction

Sea beets or wild beets [*Beta vulgaris* ssp. *maritima* (L.) Arcang.] are wild relatives of cultivated sugar beet. Sea beets have a large area of distribution along the Mediterranean and North Atlantic coasts, from the British Isles to the Canary Islands (Doney et al., 1990). A difference in vernalization requirements and hence flowering time related to latitude can be observed across their area of distribution (Van Dijk et al., 1997). While populations from the Mediterranean Sea and the Atlantic coast up to Brittany are observed to have little to no vernalization requirement, Northern Atlantic populations exhibit a low frequency of flowering within the first year (Van Dijk et al., 1997). This facilitates the use of Northern Atlantic populations in breeding.

The breeding gene pool of sugar beet is narrow, and it is considered to lack sufficient genetic variation to cope with stress (Fénart et al., 2008; Panella et al., 2020; Veloso et al., 2021). Sea beet populations possess a high level of genetic diversity with useful traits that can be harnessed to improve crop resilience, disease resistance or tolerance against abiotic stress (Ribeiro et al., 2016; Panella et al., 2020; Ascarini et al., 2021; Veloso et al., 2021; Sandell et al., 2022). At the same time, the low linkage disequilibrium observed in sea beet populations due to many generations of outcrossing generally allows for the direct use of these populations in association mapping (Capistrano-Gossmann et al., 2017). Hence, sea beet populations hold great potential for crop improvement in breeding programs.

There is an increasing number of studies on the genetic diversity of sea beet populations. Some of these studies focus on assessing the diversity based on morphological characteristics of sea beet populations sampled along the coasts of England and Ireland (Doney et al., 1990), Northern Italy (Bartsch and Schmidt, 1997), in Egypt (Abdelhameed et al., 2024) or Tunesia (Ben Mahmoud et al., 2025).

Other studies focus on the genetic diversity of sea beet populations. Sea beet populations along the coast of Dorset (Poole Harbour and adjacent coast) were analyzed for seven polymorphic isozyme loci and six polymorphic RFLP loci (Raybould et al., 1996, 1998). A total of 300 individuals across 54 populations from France was analyzed using five single-copy RFLP and one microsatellite marker (Desplanque et al., 1999). Twelve Danish, two Swedisch, one French, one Italian, one Dutch and one Irish population of sea beets were screened with eight microsatellite markers to evaluate genetic variation and gene flow of around 25 individuals per population (Andersen et al., 2005). Sea beet populations alongside the coast of the English Channel in Guernsey, Jersey and Northern France were analyzed with seven microsatellite loci, with sample sizes ranging from 18 to 62 individuals per population (Fievet et al., 2007). Eleven sea beet populations, each consisting of 27 to 49 individuals were sampled along the Channel French coastline and genotyped with five nuclear microsatellite loci (Fénart et al., 2008). Three Portuguese sea beet populations were evaluated based on six microsatellite markers (Ribeiro et al., 2016). Fourteen plants from each of eleven Madeiran sea beet populations were characterized using morphological descriptors and eight polymorphic Simple Sequence Repeats markers (Ascarini et al., 2021). Wild beet populations from fourteen locations in western Iberia, the Azores and Madeira islands were analyzed based on 9 to 35 plants per populations using six SSR loci amplifying a total of 100 alleles (Veloso et al., 2021). While these studies provide a first insight into the diversity of sea beet populations, they rely on either a limited number of individuals per population, a small set of markers, or both. This limits the possibility of generalizing the results.

Few newer studies analyze sea beet accessions from gene banks. 1,054 *Beta vulgaris* ssp. *maritima* accessions were analyzed with 4,436 DArT markers (Andrello et al., 2016). Around 240 sea beet accessions were analyzed with short-read sequencing data to study genomic relationships within the genus *Beta* (Sandell et al., 2022; Felkel et al., 2023). While these analyses are based on multiple markers or even sequencing data, they are based on one or few plants per accession and, hence, cannot capture the full diversity of the corresponding sea beet populations or give insight into their structure.

While most of these studies have compared populations from different geographic origins, a comprehensive diversity analysis of sea beet populations using large sample sizes per population and high-density markers has yet to be conducted.

The aim of this study is to deepen the understanding of the diversity and genetics of sea beet populations to enable their use as a source of genetic variation in sugar beet breeding. While genetic diversity is an indicator of the potential of a sea beet population to contribute new, valuable variation, the structure of a population affects how this variation can be used in breeding. For this purpose, three sea beet populations form the Northern Atlantic coast were chosen due to their predominantly biannual lifeform and analyzed with 16,201 SNP markers. Our objectives were to (1) analyze the genetic diversity of these populations, (2) assess their population structure and detect possible subpopulations, and (3) evaluate the implications for their use in sugar beet breeding, particularly concerning their suitability for direct trait mapping through association mapping.

## 2 Materials and methods

### 2.1 Plant material

**Figure 1:**
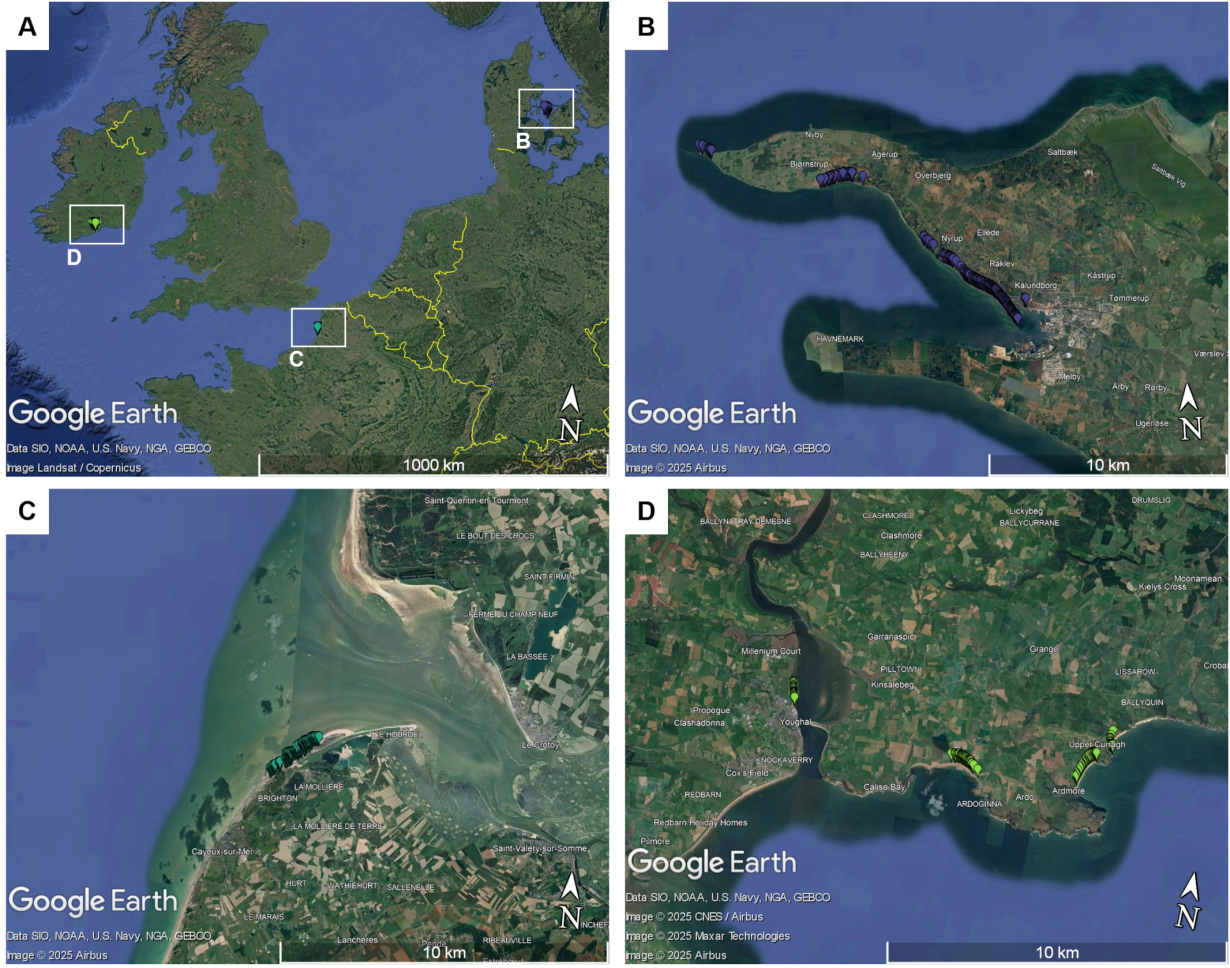
**(A)** Map showing the geographic locations of the three collected sea beet populations in northern Europe: **(B)** Denmark, **(C)** Ireland, and **(D)** France. Each marker represents the geographical location of one sampled sea beet. The maps were created using Google Earth Pro.

**Figure 2:**
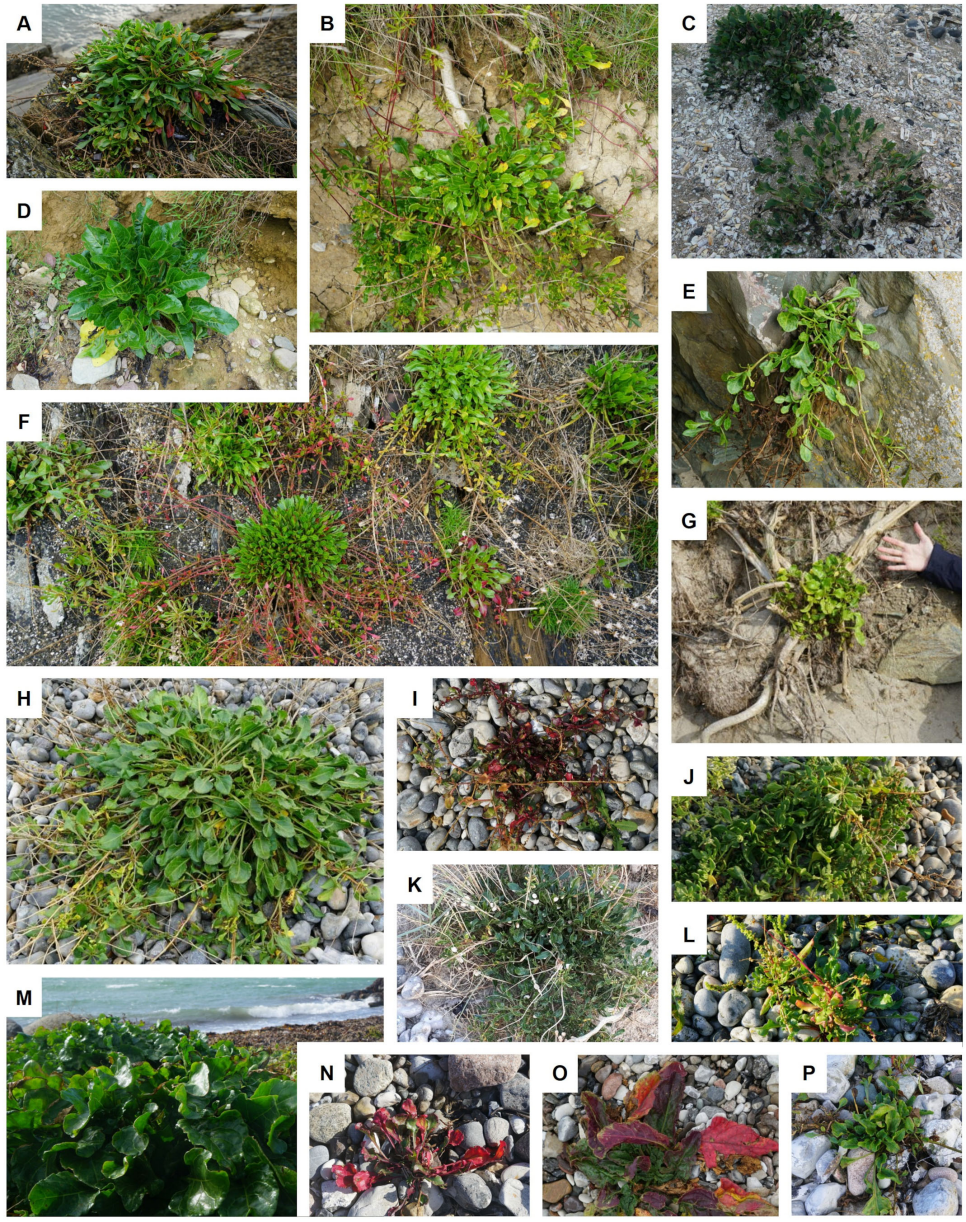
Pictures of the *Beta vulgaris* ssp. *maritima* plants found in the sampled regions: **(A–G)** Ireland, **(H–L)** France, and **(M–P)** Denmark. Not all plants shown were sampled and are included in analysis (due to seed availability). The pictures demonstrate the diversity of phenotypes within the sampled regions.

This study is based on three wild beet populations from the coast of the Northern Atlantic Sea. Seeds were sampled from the populations in their natural habitat along coastal regions in Denmark, France and Ireland. All three populations were collected from a rather small stretch along the coastline: France ~2km, Denmark ~16km, Ireland ~11km. All populations consisted of >1000 individuals. Details on the locations of the populations are given in **Figure 1**. Populations from these regions have already been described to some extent in other studies (Andersen et al., 2005; Fénart et al., 2008; Capistrano-Gossmann et al., 2017). Plants were found in very different types of habitat, ranging from edges of sandy beaches, gravel areas or even rocky cliffs. Pictures were taking during sampling. Some examples are shown in **Figure 2**, demonstrating the phenotypic diversity of the collected material.

### 2.2 Genetic data

One random offspring per sampled wild beet was grown and plant DNA was extracted using silica-membrane technology following the NucleoSpin^®^ 96 Plant II protocol from Macherey-Nagel. The samples were fingerprinted using a proprietary Illumina SNP array of KWS SAAT SE & CO KGaA. For Denmark N = 388 were genotyped, for France N = 509, and for Ireland N = 466. This resulted in a total of 1,363 genotypes across the three populations. Markers with more than two alleles and more than 1% missing values as well as all markers that were monomorphic across all three populations were discarded. All markers remaining after quality control were heterozygous in at least one individual of one population, resulting in some markers being monomorphic across one or two populations (**Table 1**). This resulted in a set of 16,201 SNP markers, that were used consistently across all datasets, enabling a comparison of results across the three populations. The markers span the entire sugar beet genome across nine chromosomes for a total length of 625 cM. The average genetic distance between markers was 0.0386 cM with a maximum of 1.8834 cM.

**Table 1:** Characteristics of the genetic data of the analyzed populations after quality control.

Population	Genotypes	Monomorphic markers	Polymorphic markers	Monomorphic only in this population	Polymorphic only in this population
Denmark	388	1,073	15,128	524	163
France	509	1,476	14,725	942	178
Ireland	466	668	15,533	264	308

All populations were analyzed using the same set of 16,201 markers. All markers remaining after quality control were heterozygous in at least one individual of one population, resulting in some markers being monomorphic across one or two populations.

### 2.3 Data analysis

All analysis were conducted using R version 4.0.5 (R Core Team, 2021).

#### 2.3.1 Linkage disequilibrium

Linkage disequilibrium (LD) analysis was performed for each chromosome by computing r^2^ values for all pairwise marker comparisons. The correlation coefficient (r^2^) was calculated using the st.calc.ld function of SelectionTools (v 23.1; https://population-genetics.uni-giessen.de/~software/) which contains the simulation routines of software Plabsoft (Maurer et al., 2008).

#### 2.3.2 Genetic diversity estimates

The observed heterozygosity $H_{O_{i}}\;$ was calculated for each individual by dividing the number of heterozygous markers by the sum of all heterozygous and homozygous markers $\left(H_{O_{i}}=\;\frac{N_{Het_{i}}\;}{N_{Het_{i}}+\;N_{Hom_{i}}}\;\right)$ . Markers with missing information for the respective individual were not considered.

The average expected heterozygosity over all loci is an estimate of the extent of genetic variability in the population. It was calculated over all 16,201 marker loci using SelectionTools. For each locus, the function st.plot.gene.diversity calculates the expected heterozygosity by subtracting the expected frequencies of homozygotes at the locus from 1 ( $H_{E_{i}}=1-\;p_{i}^{2}-\;q_{i}^{2}$ ), with *p_i_* and *q_i_* being the frequency of the two alleles at locus i. The operation was repeated for all loci. The expected heterozygosity ranges from 0 to 1 and is maximized when there are many alleles at equal frequencies. For plotting, haploblocks were built using the function st.def.hblocks based on ten adjacent markers each.

The minor allele frequency was calculated as the frequency of the less common SNP allele within each population.

Testing genetic markers for Hardy-Weinberg equilibrium was performed using a Chi-square test for goodness-of-fit as the classical test for HWE (Crow and Kimura, 1970; Graffelman, 2015), with the R package HardyWeinberg (v. 1.7.8; Graffelman, 2015) at a significance level of 0.05. Ternary plots for three-way genotypic compositions (AA, AB, BB) of all marker loci were generated for each population and also for subpopulations of France and Ireland. The assignments to subpopulations were based on the calculated admixture coefficients for the inferred number of optimal clusters per population. For the population from Ireland, Hardy-Weinberg equilibrium was also calculated for subpopulations assigned based on geographic origin.

#### 2.3.3 Genetic distances and population structure

Genetic distances were calculated as modified Roger’s distance for each possible pair of individuals using SelectionTools. A hierarchical cluster analysis was performed on these genetic distances using the hclust() function of the R package stats. The heatmap with the attached dendrogram was then produced using the R package ComplexHeatmaps (v 2.15.4; Gu et al., 2016; Gu, 2022).

Principal coordinate analysis was performed to investigate the relationship among the populations based on the previously estimated genetic distances using the cmdscale function in R (R Core Team, 2021).

As a complementary approach, the assignment of individuals from the three sea beet populations to genetic clusters was inferred using R package LEA (v 3.2.0; Frichot and François, 2015). The snmf function estimates population genetic structure from the genotype matrix using sparse Non-Negative Matrix Factorization algorithms and provides a least-squares estimate of ancestry proportions rather than maximum likelihood estimates (Frichot et al., 2014; Frichot and François, 2015). Admixture coefficients for each individual were estimated for K = 3 to 10 based on allelic data of 16,201 SNPs for all three populations. For every value of K, 100 repetitions were carried out. The estimated individual admixture coefficients for the run with the lowest cross entropy value was plotted using the barplot function of R package ggplot2 (v 3.4.4; Wickham, 2016).

To infer the number of major components within the data for up to K = 10 clusters, kmeans as clustering method was used (R package factoextra v 1.0.7; Kassambara and Mundt, 2020). The plot represents the variance within the clusters, which decreases as K increases. The Elbow method was used to select the number of clusters by minimizing the within-cluster sum of squares. With this graphical approach, the within-cluster sum of squares is plotted against the different K-values. The optimal K is identified at the point where the graph bends, forming an elbow.

To infer the number of subpopulations in Ireland and France, admixture coefficients were estimated based on the results of the analysis across all three populations for K = 2 to 5 in Ireland and for K = 2 to 4 in France. The optimal number of clusters within each of the populations was inferred also using kmeans clustering and the Elbow method.

## 3 Results

### 3.1 Linkage disequilibrium

**Figure 3:**
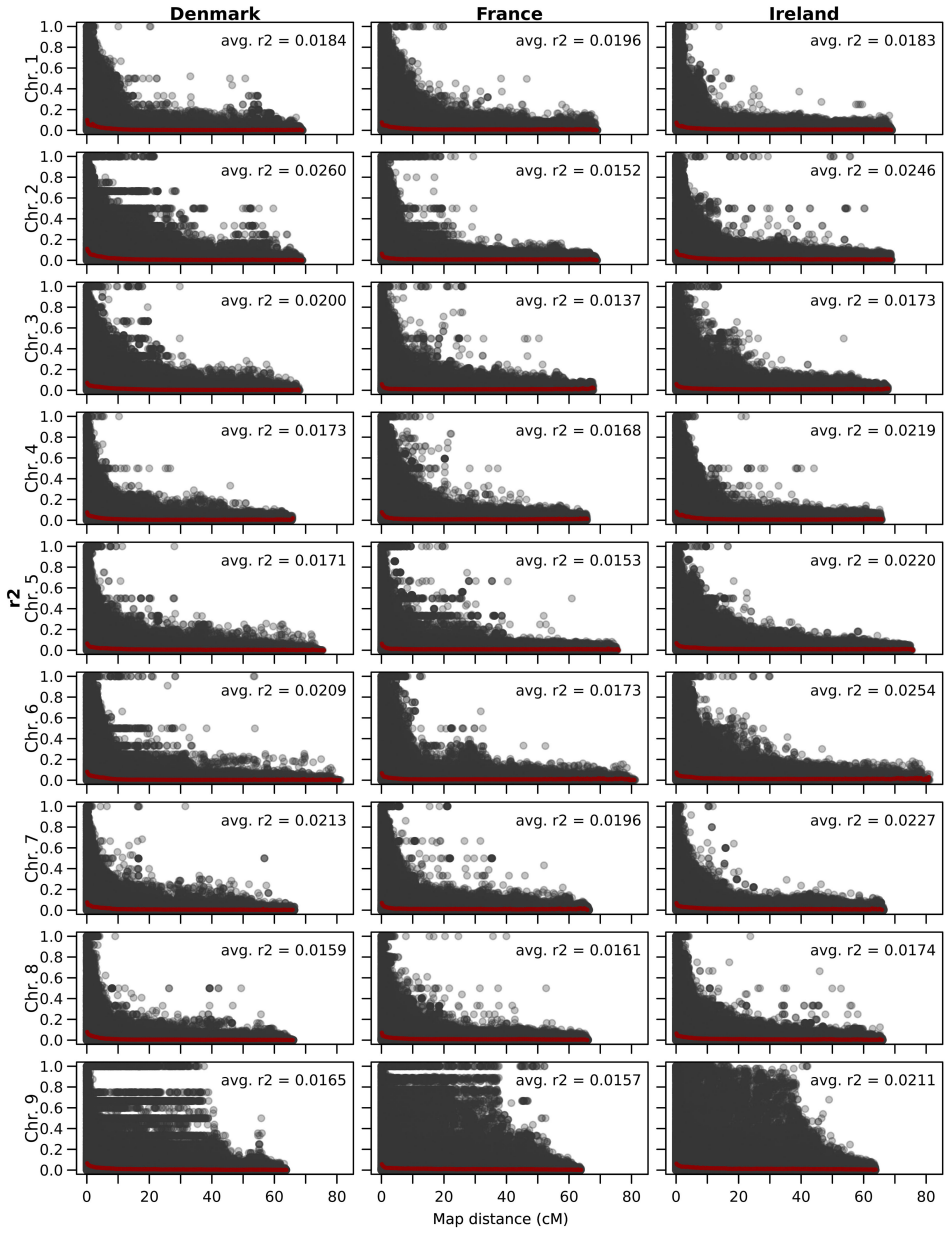
Scatter plot showing the linkage disequilibrium decay per chromosome of all three sea beet populations. The genetic distance (cM) on the x-axis is plotted against estimates of the linkage disequilibrium correlation coefficient (r^2^) for pairs of markers on the y-axis. In red, a trend line is shown and the average correlation coefficient is given for every chromosome.

The linkage disequilibrium, calculated as the average pairwise correlation coefficient (r²), is consistently low across all three analyzed populations and throughout all chromosomes (**Figure 3**). The lowest value was observed in France (0.0178), followed by Ireland (0.0190), with Denmark exhibiting the highest value (0.0194). The average linkage disequilibrium varies across chromosomes, and the chromosome with the highest or lowest values differs between populations. Overall, the lowest LD was observed on chromosome 3 in France (0.0137), while the highest was recorded on chromosome 2 in the Danish population (0.0260).

### 3.2 Heterozygosity

**Figure 4:**
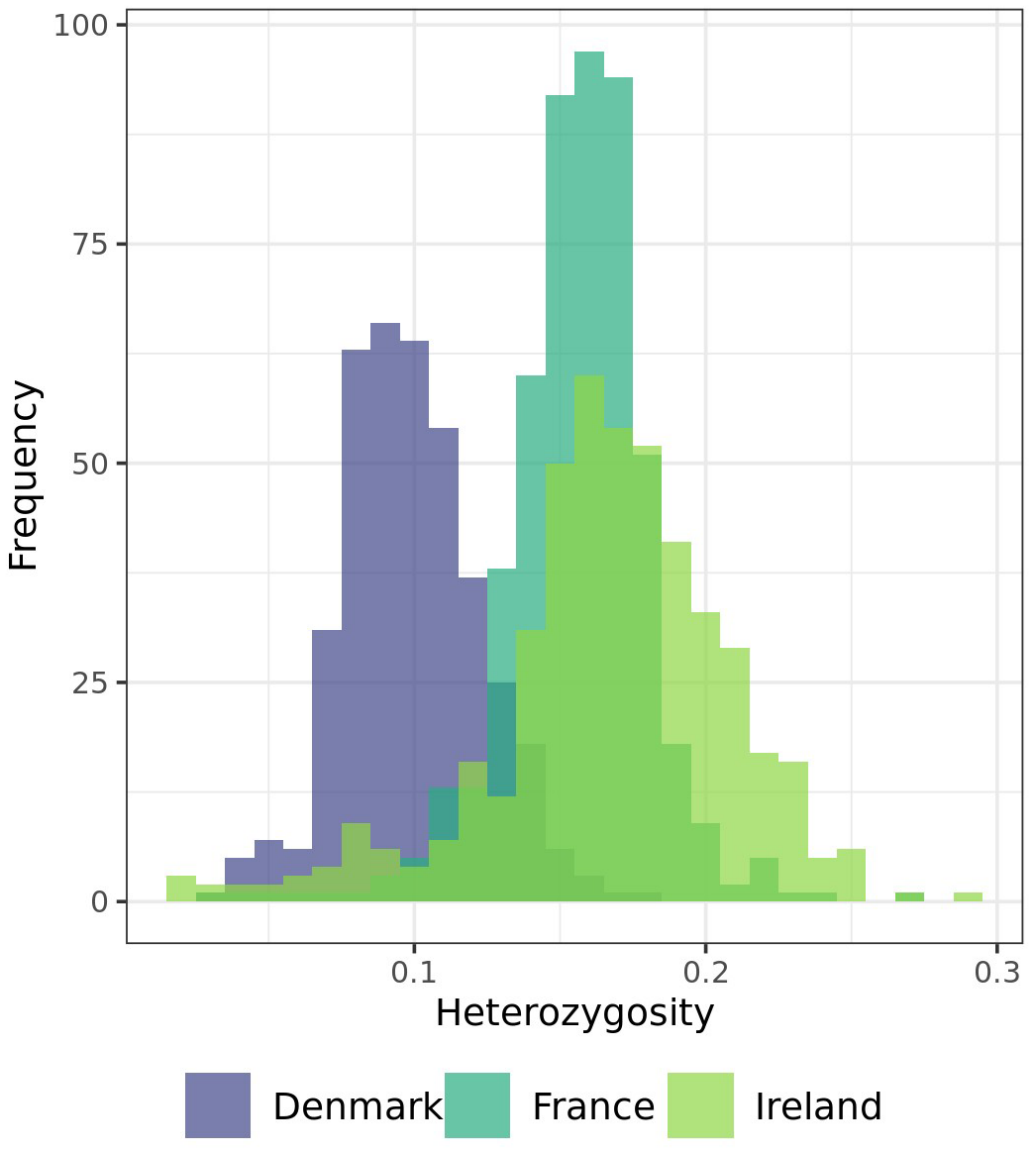
Distribution of individuals of each population by their heterozygosity (y-axis shows the heterozygosity of the individuals in %).

Observed heterozygosity is low across all individuals analyzed within this study (**Figure 4**). None of the individuals within all three populations showed a heterozygosity of more than 30%. On average 9.89% of the analyzed markers in the population from Denmark, 15.58% on average in the population from France, and 16.68% on average in the population from Ireland were heterozygous. While the standard deviation of minor allele frequencies is relatively low in the Danish (2.35%) and French (2.50%) populations, the Irish population exhibits greater variability, with a standard deviation of 4.12%.

**Figure 5:**
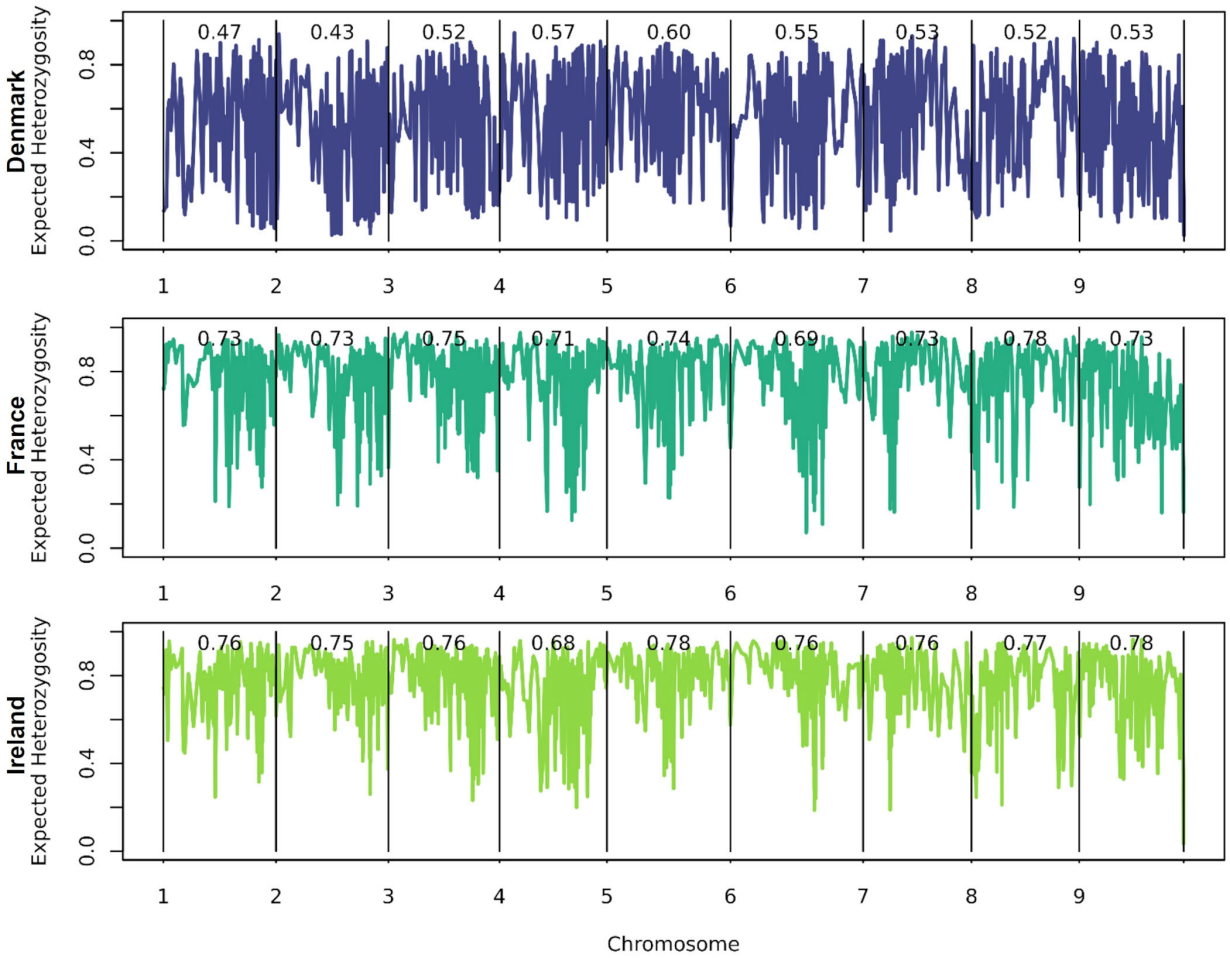
Average expected heterozygosity over all loci as an estimate of the extent of genetic variability in the population calculated over all 16,201 marker loci. Haploblocks were built based on 10 adjacent markers each.

Expected heterozygosity (**Figure 5**) is higher than the observed heterozygosity. Sea beets from France and Ireland exhibit greater expected heterozygosity compared to those from Denmark. While in Denmark the average expected heterozygosity ranges from 0.43 on (chr. 2) to 0.60 (chr. 5), in France from 0.69 (chr. 6) to 0.78 (chr. 8), and in Ireland from 0.68 (chr. 4) to 0.78 (chr. 5 and chr. 9). Hence, the variation between chromosomes is higher within the population from Denmark. Different regions and different chromosomes seem to be under selection within the different populations. As can be seen also in **Table 1**, each population has a certain number of exclusive polymorphisms.

### 3.3 Minor allele frequencies

**Figure 6:**
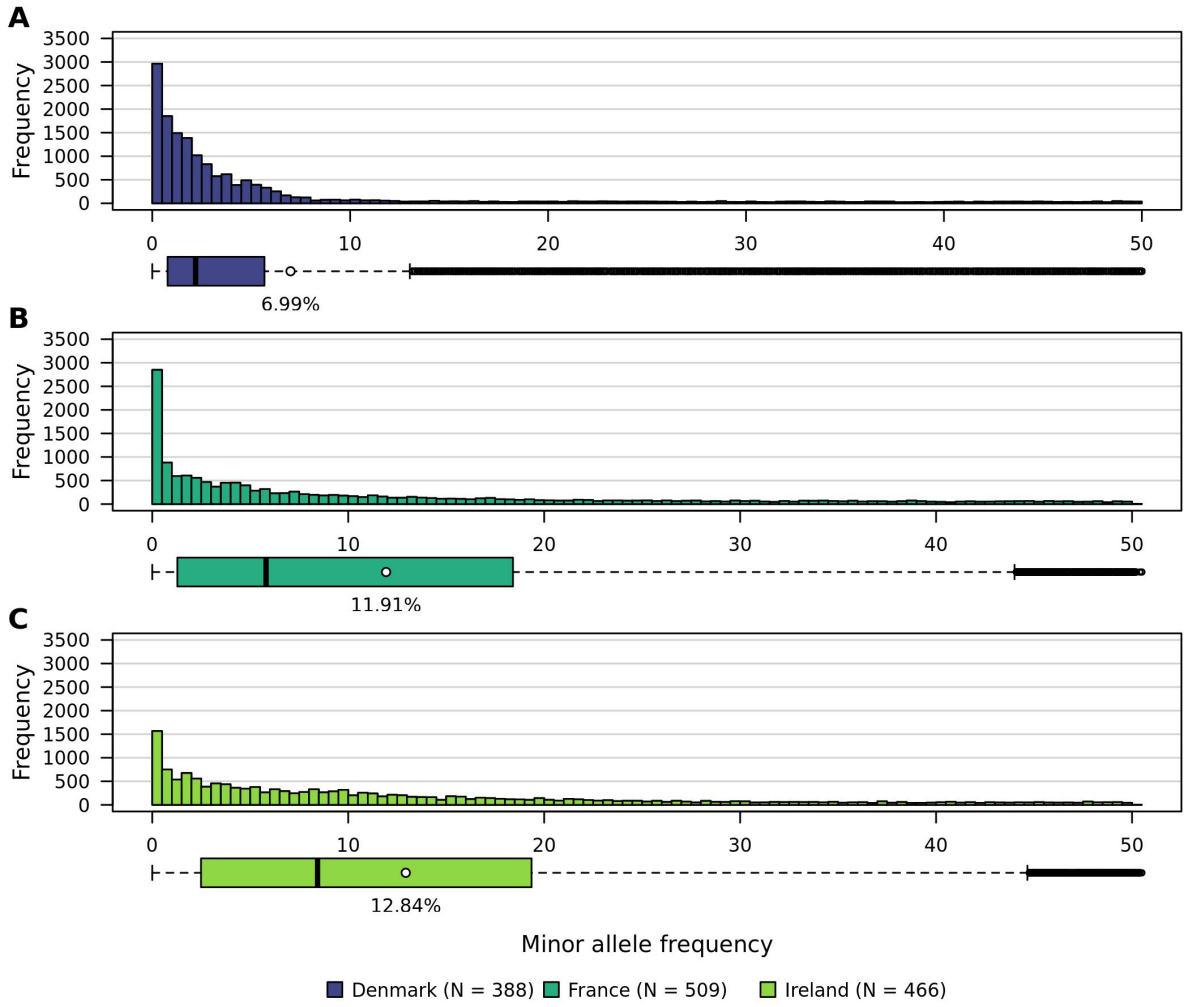
Histograms showing the distribution of minor allele frequencies within all three sea beet populations. **(A)** Denmark, **(B)** France, and **(C)** Ireland.

Minor allele frequencies observed within the three analyzed populations are low (**Figure 6**). On average they lie between 6.99% in Denmark, 11.91% in France, and 12.84% in Ireland. While the distributions of minor allele frequencies in the Irish and French populations are similar, the Danish population exhibits a distinct pattern. The interquartile ranges for France and Ireland are comparable, whereas Denmark has a noticeably smaller interquartile range. Additionally, the variance (132.21) and standard deviation (11.49%) within the Danish population are lower than those observed in the French (187.67; 13.70%) and Irish (170.43; 13.05%) populations.

### 3.4 Hardy-Weinberg equilibrium

**Figure 7:**
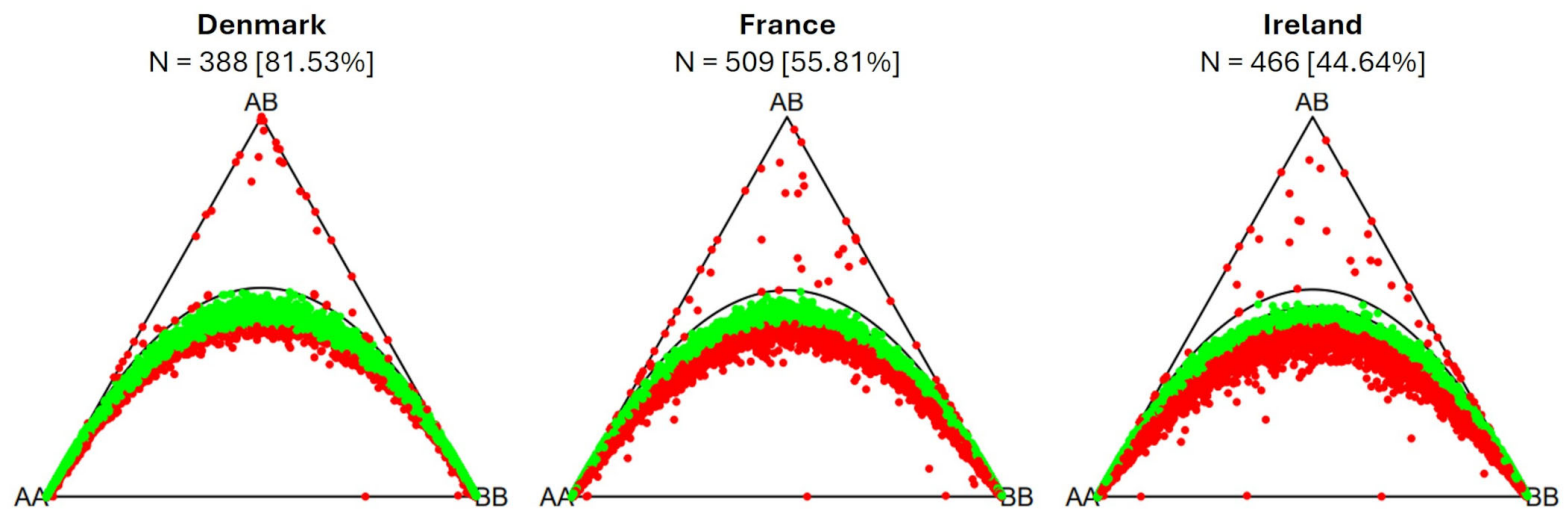
Ternary plots for three-way genotypic compositions (AA, AB, BB) of all 16,201 SNP marker loci. The parabolas within the plot represent the acceptance region corresponding to the Chi-square test for Hardy-Weinberg equilibrium. The (non-)significance of the test can be inferred from the position of the markers in the ternary plot. Significant markers are indicated by red points, non-significant markers by green points. Significance level is 0.05. The number of individuals within each subpopulation (N) is depicted above each plot. Values in brackets determine the percentage of markers in Hardy-Weinberg equilibrium.

The percentage of markers in Hardy-Weinberg equilibrium varies across populations (**Figure 7**). The highest percentage of markers in Hardy-Weinberg equilibrium was observed within the population from Denmark (81.53%). The population from France has fewer markers in Hardy-Weinberg equilibrium (55.81%), while the population from Ireland has the lowest percentage of markers in Hardy-Weinberg equilibrium (44.64%). In both populations a heterozygote deficiency (excess of homozygotes) can be observed.

### 3.5 Principal coordinate analysis

**Figure 8:**
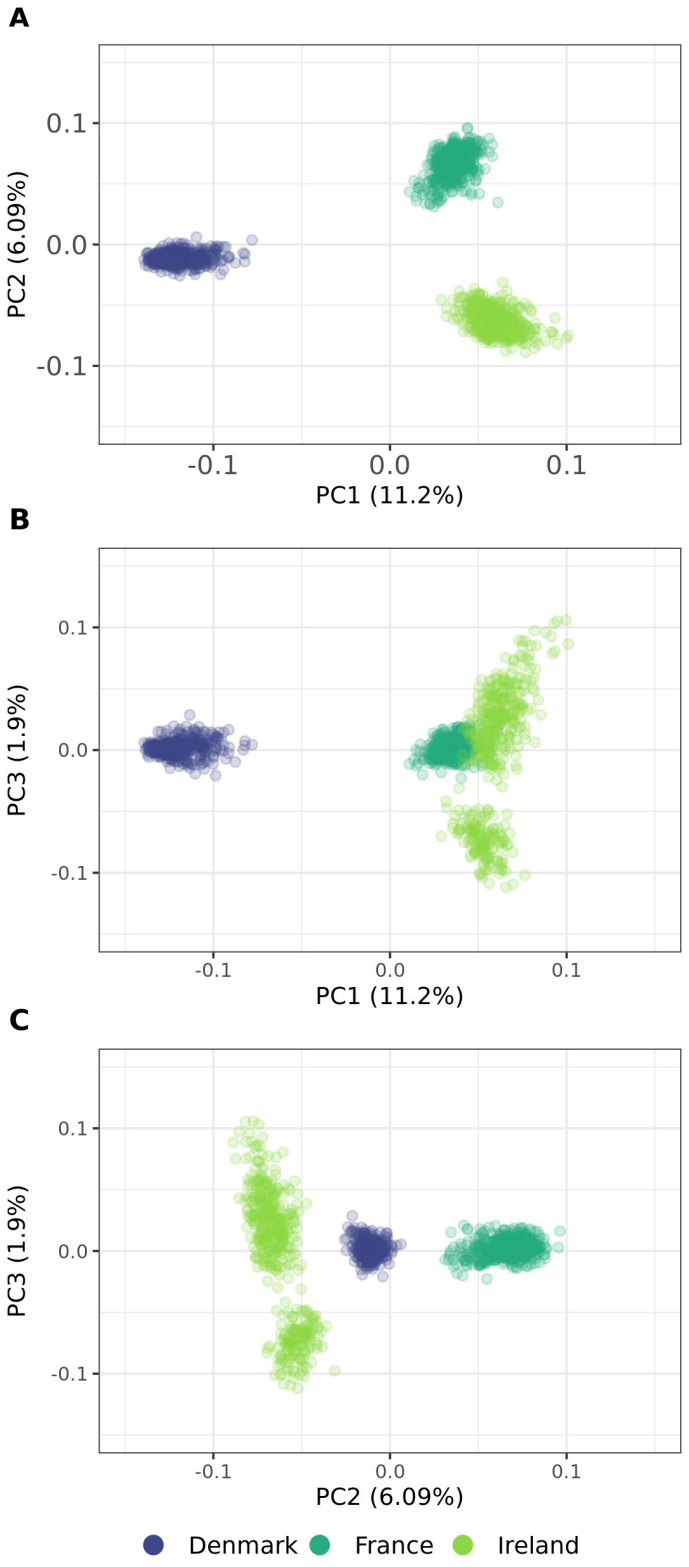
**(A–C)** Principal coordinate analysis plots of 1,363 individuals from three sea beet populations. Genetic distances are calculated as modified Roger’s Distance based on 16,201 SNP marker loci. PC1, PC2 and PC3 are the first, second, and third principal coordinate, respectively, and the values in parentheses refer to the proportion of variance explained. Colors are assigned according to population in all graphs.

Principal coordinate analysis was conducted to investigate relationships among the populations and their individuals. The first three coordinates collectively accounted for 26.59% of the total variation. The first two axes explained 11.20% and 6.09% of the total variation, respectively, and identified three distinct genetic groups (**Figure 8**). The analysis revealed that genetic diversity between populations is considerably greater than within them. The three populations were clearly differentiated, as observed primarily in PC1 and PC2. In contrast, PC3 indicated some genetic admixture between Denmark and Ireland, as well as France and Ireland, while populations from France and Denmark remained distinctly separate (**Figure 8**). Furthermore, whereas individuals from Ireland exhibited a more dispersed clustering pattern along PC1 and PC2 compared to other populations, PC3 revealed the presence of subpopulations within the Irish population.

### 3.6 Genetic distances

**Figure 9:**
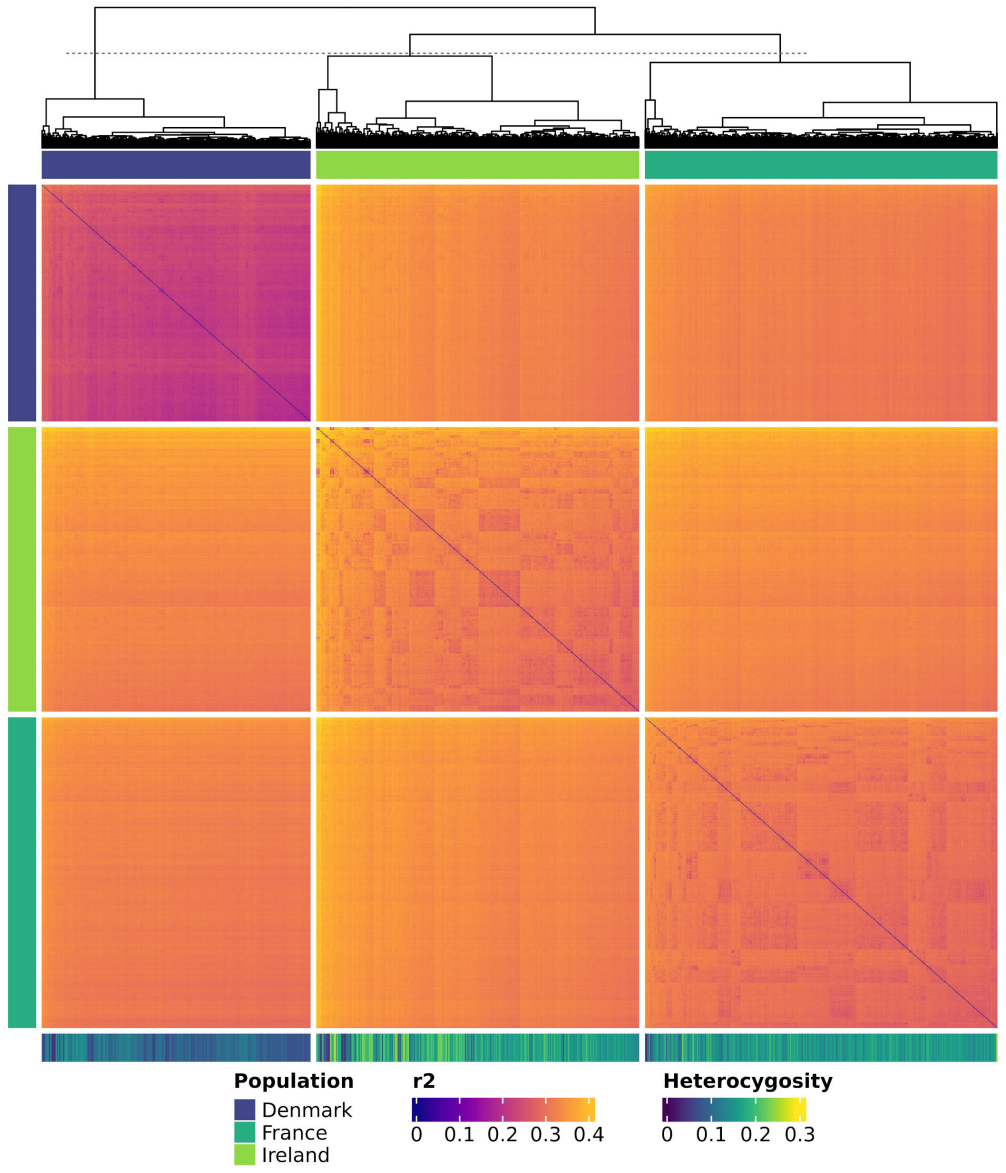
Heatmap showing genetic distances among all individuals. Genetic distances are calculated as modified Roger’s Distance. Individuals are colored by population and clustered according to the dendrogram. Each row and each column represent a genotyped individual. The colored bars at the top and the left of the heatmap show the corresponding population of the individuals. The horizontal bar at the bottom shows the heterozygosity of each individual.

The heatmap and dendrogram of genetic distances (**Figure 9**) indicate a genetic separation of the Danish population from the Irish and French populations. The latter two exhibit closer genetic relatedness. Additionally, individuals within the Danish population are more genetically homogeneous compared to those in the other two populations. In contrast, individuals from the French, and to an even greater extent those from the Irish population, show a higher genetic divergence within their population. This pattern is evident in both the dendrogram and the genetic distance measurements. The average genetic distance, estimated as modified Roger’s distance, was 0.231 within the Danish population, 0.304 within the French population, and 0.324 within the Irish population. Notably, the average modified Roger’s distance across all three populations was 0.320, slightly lower than the value observed within the Irish population.

### 3.7 Admixture coefficients

**Figure 10:**
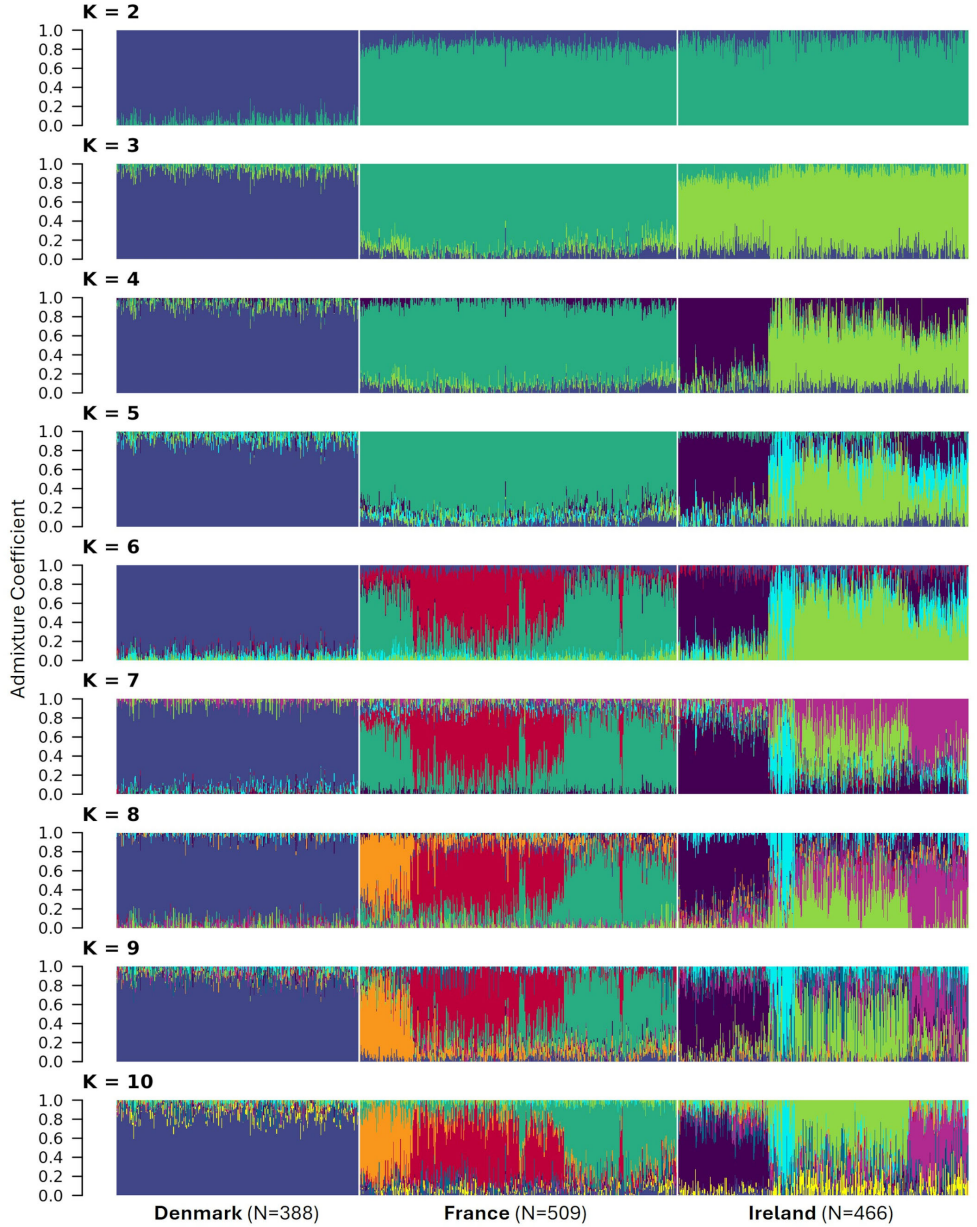
Assignment of 1,363 individuals from three sea beet populations to genetic clusters. Admixture coefficients for K = 2 to 10 were computed based on allelic data of 16,201 SNPs. Each individual is represented by a vertically stacked column indicating the proportions of ancestry in K constructed ancestral populations. Populations are separated by whilte vertical lines based on geographic origin.

As a complementary approach, admixture coefficients for each individual were calculated for K = 2 to 10 (**Figure 10**). Admixture analysis also revealed clear genetic differentiation among the three populations. Accordingly, k-means clustering identified K = 3 as the most likely number of clusters within the dataset. Furthermore, the populations from France and Ireland exhibited greater genetic similarity to each other than to the Danish population as can be seen for K = 2. Despite being sampled across the largest geographical range, individuals from Denmark demonstrated a high degree of genetic homogeneity, with no detectable subpopulations even for K = 10. In contrast, substructure was observed in the French population for K ≥ 6. K-means clustering estimated the optimal number of clusters for this population to be K = 3 (results not shown). The Irish population, however, exhibited pronounced genetic substructure, which is consistent with its deviation from Hardy-Weinberg equilibrium. The most probable number of subpopulations within the Irish dataset was estimated to be K = 4.

## 4 Discussion

### 4.1 Linkage disequilibrium

The low linkage disequilibrium observed in sea beet populations allows for the direct use of these populations in association mapping, rather than constructing biparental populations for quantitative trait locus analysis. Many generations of outcrossing result in a low linkage disequilibrium within these populations. The calculated average pairwise correlation coefficient (r^2^) regarding all chromosomes is lowest in France, followed by Denmark and highest in Ireland. Overall, the linkage disequilibrium within all three analyzed populations is very low across all chromosomes (**Figure 3**). This is expected for sea beet populations of outcrossing species which have undergone many generations of random mating and has been described in sea beet populations also in other studies (Fievet et al., 2007; Capistrano-Gossmann et al., 2017). Lower linkage disequilibrium allows for higher resolution in detecting regions associated with traits (Garnier-Géré and Chikhi, 2013), as demonstrated by the successful mapping of the Rhizomania resistance gene Rz2 within the sea beet population sampled near Kalundborg in Denmark (Capistrano-Gossmann et al., 2017). Therefore, while developing biparental populations for QTL analysis can be time-consuming and costly, sea beet populations with low linkage disequilibrium are highly suitable for association mapping due to their inherited high resolution.

### 4.2 Heterozygosity

Observed heterozygosity is low across the three analyzed sea beet populations, with none of the individuals showing a heterozygosity of more than 30% (**Figure 4**). This is contrary to most of the literature, where *Beta vulgaris* ssp. *maritima* (L.) Arcang. is described as a naturally outcrossing species with a high degree of self-incompatibility and hence usually high levels of heterozygosity within the populations (Hautekeete et al., 2020; Veloso et al., 2021; Felkel et al., 2023). While the observed heterozygosity is low, the average expected heterozygosity is high (**Figure 5**). Expected heterozygosity estimates the probability that two alleles randomly chosen from a population will be different, indicating genetic diversity. Expected heterozygosity is highest within the populations from France and Ireland and lowest the population from Denmark (**Figure 5**). Lower observed heterozygosity compared to expected heterozygosity, as found in this study, suggests inbreeding or population structure. Certain genome regions appear to be under selection within one population, with specific alleles unique to each population (**Figure 5**, **Table 1**). Further analyses would be required to determine the cause of selection within the populations, which is beyond the scope of this study. Nevertheless, the high expected heterozygosity indicates a substantial genetic diversity within these populations.

### 4.3 Minor allele frequency

The minor allele frequencies observed within the analyzed populations are low, which corresponds to the low heterozygosity observed in this study. The high genetic diversity of sea beet populations may also result in many alleles being present at low frequencies. Minor allele frequencies observed within the three analyzed populations on average lie between 7.0% in Denmark, 11.9% in France, and 12.9% in Ireland (**Figure 6**). Low minor allele frequencies can pose challenges for association mapping, as rare alleles are more likely to be observed in heterozygotes rather than homozygotes (Bernardo, 2010), reducing the statistical power to detect associations. Additionally, literature shows that low minor allele frequencies can lead to an increased rate of false positives (Tabangin et al., 2009). This occurs because the sample size of individuals carrying the rare allele is small, leading to higher variability and less reliable statistical inference.

### 4.4 Genetic diversity

Genetic diversity was overall highest in the Irish population, while the Danish population exhibited the lowest phenotypic and genetic variation. While high genetic diversity is beneficial, it also requires careful sampling and appropriate statistical analysis to ensure that the associations detected are accurate and not due to random chance and to effectively utilize high genetic diversity in association mapping studies. Firstly, to capture the full extent of genetic diversity within a population, it is crucial to sample a sufficiently large number of individuals within the population. Given the high genetic diversity of the French and especially Irish populations, ensuring adequate sampling sizes will be essential to comprehensively represent their genetic variation. On the other hand, studies should incorporate a substantial number of entries to ensure proper representation of low-frequency alleles within the analysis. This enhances the statistical power and reliability of the associations detected. Additionally, sophisticated statistical models are necessary to accurately detect true associations in populations with high genetic diversity (Santure and Garant, 2018; Otyama et al., 2019). These models need to account for the low frequency of rare alleles and help reduce the variability and improve the reliability of the results. Especially for the Danish population with the low minor allele frequency appropriate statistical models and sufficient study sizes will be crucial. Despite these challenges, sea beet populations can be valuable for association mapping studies when a sufficient sample size and appropriate statistical models for analysis are employed. Appropriate measures must be tailored to the genetic diversity present within each population. Based on the observed high genetic diversity, the Irish population appears to have the most potential to directly map traits by association mapping and to contribute new variation to breeding programs.

### 4.5 Hardy-Weinberg equilibrium

Among the three analyzed sea beet populations, the population from Denmark shows the highest percentage of markers in Hardy-Weinberg equilibrium (81.53%; **Figure 7**). This corresponds to its low minor allele frequencies and low heterozygosity. The population from France has fewer markers in Hardy-Weinberg equilibrium (55.81%), while the population from Ireland has the lowest percentage of markers in Hardy-Weinberg equilibrium (44.64%). In both populations a heterozygote deficiency (or excess of homozygotes) can be observed. Other studies also found deviations from Hardy-Weinberg equilibrium caused by deficiency in heterozygotes within some sampled sea beet populations (Andersen et al., 2005; Fievet et al., 2007).

This deviation from Hardy-Weinberg equilibrium in the French and Irish populations suggests the presence of non-random mating or population substructure. This can occur, when individuals are more likely to mate with others that are genetically similar to them, also called positive assortative mating (Crow and Kimura, 1970; Garnier-Géré and Chikhi, 2013). Under these conditions a certain amount of inbreeding and, hence, more homozygote loci than expected under Hardy-Weinberg, will be observed (Garnier-Géré and Chikhi, 2013; Chen et al., 2017). Individuals may for example be more likely to mate, if they are located geographically close together or have a similar time of flowering (Breese, 1956; Fox, 2003). A large range in flowering time and in some areas even perennial growth habit has been observed during the sampling of populations within this study (**Figure 2**) as well as in others (Doney et al., 1990; Bartsch and Schmidt, 1997). This may be one explanation for the observed deviation from Hardy-Weinberg equilibrium in these populations.

Subpopulations between which there is partial or complete isolation may also occur due to other reasons such as geographic barriers or environmental factors. In such cases, each subpopulation may experience genetic drift, selection, or other evolutionary forces that cause their allele frequencies to diverge (Crow and Kimura, 1970; Chen et al., 2017). When these subpopulations are considered together, the overall heterozygosity is lower than expected under Hardy-Weinberg equilibrium. The combination of different allele frequencies reduces the proportion of heterozygotes, leading to a heterozygote deficit (Chen et al., 2017). This is also known as Wahlund effect (Crow and Kimura, 1970; Garnier-Géré and Chikhi, 2013).

As the efficiency of association mapping studies strongly depends on patterns and extent of linkage disequilibrium within in sea beet populations (Garnier-Géré and Chikhi, 2013), differences in allele frequencies between subpopulations can lead to false positives. This is caused by genetic variants appearing to be associated with a trait simply because of population stratification rather than a true causal relationship (Bernardo, 2010). Hence, deviations from Hardy-Weinberg equilibrium can reduce the statistical power to detect true associations and increase the likelihood of false positives. While this poses less of an issue within the Danish population, this may severely influence analysis of the populations from France and Ireland. Consequently, correcting for population structure is essential to avoid these confounding effects and to accurately identify genetic variants linked to traits of interest, especially in the latter populations (Bernardo, 2010; Garnier-Géré and Chikhi, 2013).

### 4.6 Genetic distances and population structure

Population structure and genetic distance of natural sea beet populations also play a crucial role in their useability for association mapping studies. The three evaluated populations from Denmark, France, and Ireland exhibit distinct genetic clusters, as evidenced by principal coordinate analysis (**Figure 8**), dendrogram and heat map (**Figure 9**), and admixture coefficients (**Figure 10**). Genetic diversity is much larger between the analyzed populations than within these according to the principal coordinate analysis (**Figure 8**). The Danish population exhibits strong genetic distinction from both the Irish and French populations, with the latter two appearing more closely related to each other within principal coordinate analysis and dendrogram.

This pattern aligns with findings from previous studies, where Danish populations show strong genetic similarity among themselves, with little population structure, and remain notably distinct from other European populations (Andersen et al., 2005; Felkel et al., 2023). This genetic distinctiveness may result from their geographic isolation at the Northern edge of the sea beet distribution range. Although Denmark is connected to other regions by waterways, physical distance likely may act as a barrier, limiting gene flow from other populations. This is also reflected in the population’s high number of monomorphic markers and the limited presence of exclusive polymorphisms (**Table 1**). Interestingly, Danish populations exhibit closer genetic relatedness to Irish populations than to French populations (**Figures 8**, **9**), a trend also reported in earlier research (Andersen et al., 2005; Andrello et al., 2016). Other studies have observed a decrease in genetic diversity from Southern to Northern Atlantic coastal regions (Leys et al., 2014; Veloso et al., 2021).

Little population structure and close genetic relatedness (estimated modified Roger’s distance = 0.231) can be observed within the Danish population (**Figures 8**-**10**). This absence of subpopulations within the Danish population, even with K = 10 (**Figure 10**) and despite sampling across a large geographical distance, indicates a high level of gene flow within this population. In contrast, the populations from Ireland and France exhibit more pronounced substructure. This is consistent with findings from other studies, which also have observed more population structure in populations from France and Ireland than from Denmark (Felkel et al., 2023).

**Figure 11:**
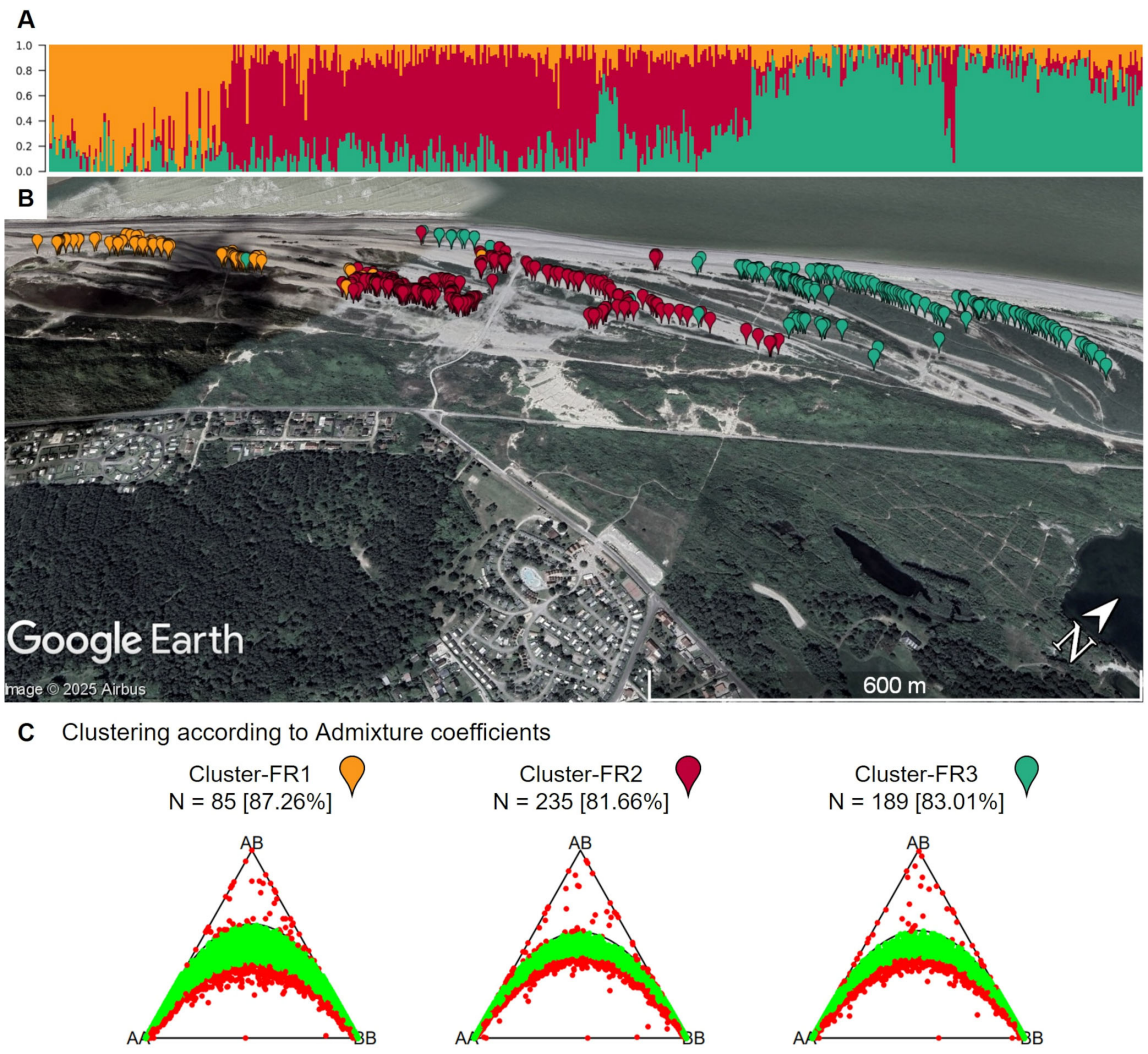
**(A)** Admixture coefficients for K = 3 for all 509 individuals from the French sea beet population. Each individual is represented by a vertically stacked column indicating the proportions of ancestry in K constructed ancestral populations. Individuals are ordered based on geographic origin **(B)** Map shows the geographic origin of the sea beets colored by assigned group according to admixture coefficients. Within the short geographic stretch, a gradient can be observed. The map was generated using GoogleEarth Pro. **(C)** Ternary plots for three-way genotypic compositions of all 16,201 SNP marker loci for the population from France divided into K = 3 clusters based on admixture coefficients. The parabolas within the plot represent the acceptance region corresponding to the Chi-square test for Hardy-Weinberg equilibrium. The (non-)significance of the test can be inferred from the position of the markers in the ternary plot. Significant markers are indicated by red points, non-significant markers by green points. Significance level is 0.05. The number of individuals within each subpopulation (N) is depicted above each plot. Values in brackets determine the percentage of markers in Hardy-Weinberg equilibrium.

The French population shows substructure, despite being sampled along a short geographic stretch (**Figure 11A**). K-means cluster analysis estimated the optimal number of clusters to be three. A clear correlation between the geographic origin and the allocated clusters can be seen (**Figure 11B**). However, the reason for the emergence of these subpopulations is not clear and would require further investigation within another study. When these subpopulations are analyzed separately, the percentage of markers in Hardy-Weinberg increases (**Figure 11C**). This confirms the presence of subpopulations.

**Figure 12:**
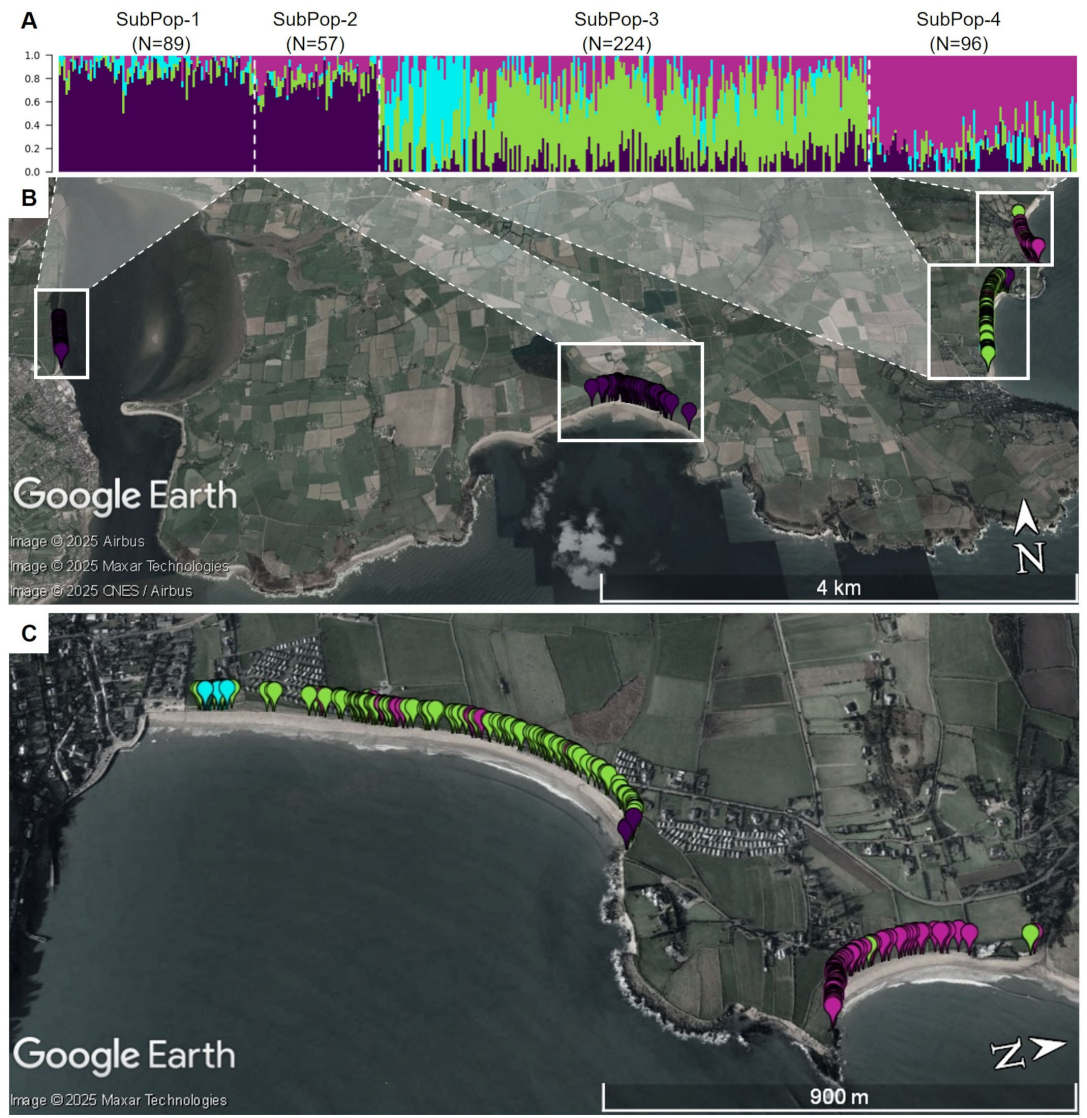
**(A)** Admixture coefficients for K = 4 for all 466 individuals from the Irish sea beet population. Each individual is represented by a vertically stacked column indicating the proportions of ancestry in K constructed ancestral populations. Populations are separated by dotted white vertical lines and ordered according to geographic origin **(B)** Map shows the geographic origin of the sea beets colored by assigned group. White dotted lines were added, to show where individuals from **(A)** are located geographically. Two of the subpopulations group together, despite being further away, whereas there is admixture within one group and within a short geographic stretch. **(C)** Shows an enlarged view of SubPop-3 and SubPop-4, turned by 90 degrees for better view. The maps were generated using GoogleEarth Pro.

**Figure 13:**
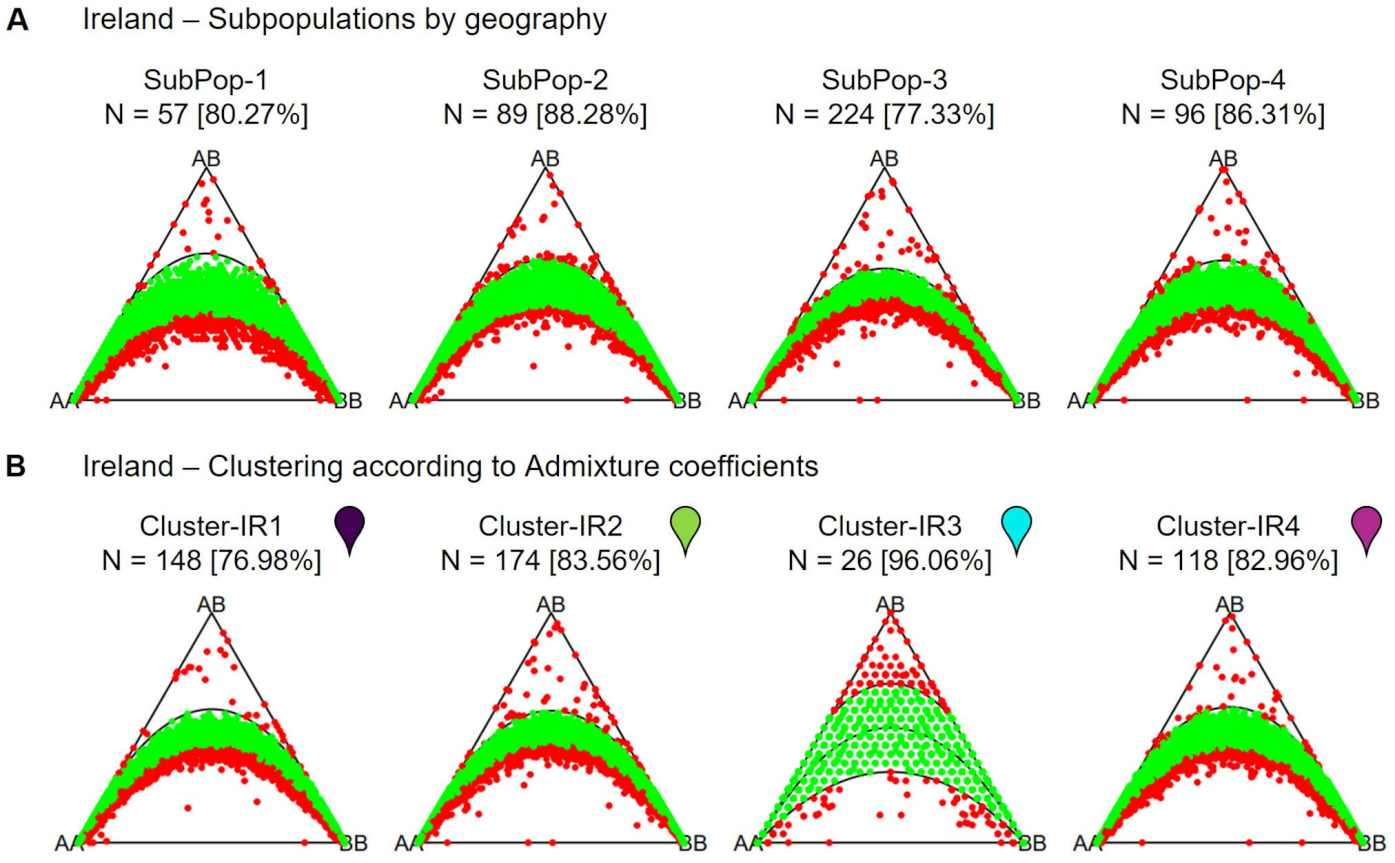
Ternary plots for three-way genotypic compositions (AA, AB, BB) of all 16,201 SNP marker loci. The parabolas within the plot represent the acceptance region corresponding to the Chi-square test for Hardy-Weinberg equilibrium. The (non-)significance of the test can be inferred from the position of the markers in the ternary plot. Significant markers are indicated by red points, non-significant markers by green points. Significance level is 0.05. The number of individuals within each subpopulation (N) is depicted above each plot. **(A)** divided into subpopulations based on the 4 geographic sampling regions, **(B)** divided into K = 4 clusters based on admixture coefficients. Colored pins represent the color of the cluster in corresponding admixture plot for K = 4 (**Figure 12**).

The Irish population also shows clear substructure, with the most likely number of subpopulations estimated to be K = 4 (**Figure 12**). This population was sampled along a coastline stretch of ~11km, interrupted by cliffs and inaccessible stretches. The population therefore consists of four geographically separated sampling sites (**Figures 1**, **12**). However, these do not constitute the four separate genetic clusters. Rather, the two sites geographically furthest apart seem to be related closest genetically (**Figure 12**). On the other hand, a strong presence of population substructure is detected in a very short stretch of coastline within one of the four sampling sites. When regarding subpopulations according to their admixture assignment, the number of markers found in Hardy-Weinberg equilibrium greatly increases (**Figure 13**). This suggests that population structure is in this case not caused by spatial distance. Strong population structure on a very local scale has also been observed in other studies and has been explained by founder events along with restricted gene flow (De Cauwer et al., 2012). The part of the Irish population with the most structure was sampled on a beach close to the town of Ardmore. Human activities and environmental constraints can have a major influence on the genetic variability of sea beet populations (Doney et al., 1990; Ascarini et al., 2021) and could have also impacted the structure of the population near Ardmore. However, this would require further research.

Population structure can significantly affect the outcomes of association mapping (Bernardo, 2010; Garnier-Géré and Chikhi, 2013). Differences in allele frequencies between subpopulations can lead to false positives caused by shared ancestry rather than actual genetic linkage (Bernardo, 2010). Hence, correcting for population structure in association studies is essential to avoid these confounding effects and to accurately identify genetic variants linked to traits of interest (Bernardo, 2010; Garnier-Géré and Chikhi, 2013). While this is a modest challenge in the population sampled in Denmark, the French and Irish population exhibit population structure that needs to be accounted for during analysis to ensure that detected associations reflect true genetic associations rather than population-related biases.

### 4.7 Geographic distances

The geographic distance across which populations were sampled did not correlate with their genetic diversity or their population structure in this study. For instance, the population from Denmark, sampled across the largest geographic distance (~16km), exhibited the lowest genetic diversity and little population structure. This population showed many monomorphic markers and few polymorphisms exclusive to it, with the lowest heterozygosity and closely related individuals. Conversely, the population from France, sampled along a shorter stretch of coastline (~2km), also had limited polymorphisms exclusive to it, but showed a higher minor allele frequency and average expected heterozygosity compared to the population from Denmark. The population from Ireland, sampled across an intermediate geographic distance (~11km), demonstrated the highest phenotypic and genetic diversity among all analyzed populations and the most pronounced population structure. Our results were in contrast to literature, where often a correlation between geographic distance and genetic distance is described (Raybould et al., 1998; Leys et al., 2014). However, these studies were based on data for few loci or pure morphological analysis. Other studies have also observed population structure on a very local scale and found populations extending over a larger geographical stretch (Doney et al., 1990; De Cauwer et al., 2012; Sandell et al., 2022) or described regions in which neighboring beets had lower genetic relatedness than beets from greater genetic distance (Raybould et al., 1996).

During the sampling of sea beet populations for their use in breeding, it is crucial to consider that genetic diversity and population structure are influenced by more than just geographic distance. Other factors, such as historical population dynamics, gene flow, and local environmental conditions, may play significant roles in shaping genetic diversity and population structure. A very localized sampling may not exclude population structure while broader sampling does not necessarily increase genetic diversity.

## 5 Conclusion

Genetic diversity and population structure of sea beet populations have implications for their use in sugar beet breeding. The populations from Denmark, France, and Ireland show different levels of genetic diversity and population structure, which present specific challenges for association mapping studies. While the Danish population’s lack of substructure and the high amount of markers in Hardy-Weinberg equilibrium simplifies association analysis, this population shows the lowest genetic diversity with many monomorphic markers and few polymorphisms exclusive to this population, despite being sampled across the largest geographic distance. The low minor allele frequency observed within the population requires larger study sizes to ensure that rare alleles are adequately represented in the study and hence to assure statistical power and reliability of the associations detected. In contrast, the populations from France and Ireland, with their higher genetic diversity and higher minor allele frequencies offer greater potential for detecting new valuable genetic variation. However, this also requires larger sampling sizes to cover this genetic variation. Additionally, the presence of subpopulations in these populations necessitates careful consideration of population structure in genetic analyses to avoid false positives and misleading conclusions. This is even more pronounced for the population from Ireland, where a strong population structure was observed at a very local scale. Despite this, these populations offer great potential when analysis is carried out correctly and population structure is accounted for appropriately.

Overall, while all three populations show genetic diversity and hence have the potential to contribute genetic variation to breeding programs, their successful use for breeding within association studies requires careful consideration of their genetic structure and diversity. The analysis of the three sea beet populations revealed the population from Ireland to have the highest phenotypic and genetic diversity among all analyzed populations. Based on this high genetic diversity, the Irish population appears to have to most potential for use to directly map traits by association mapping, provided that the challenges posed by the severe population structure can be adequately addressed within analysis.

## Data Availability

The datasets presented in this study can be found in online repositories. The names of the repository can be found below: https://github.com/lisabertram/Seabeetpopulations, 1363.
